# Supplementary material for: MetaRibo-Seq measures translation in microbiomes
Source: Nat Commun. 2020 Jun 29;11:3268. doi: 10.1038/s41467-020-17081-z (PMC7324362; doi:10.1038/s41467-020-17081-z)
Supplement: Supplementary file 10 — Supplementary Data 7 [file 41467_2020_17081_MOESM10_ESM.zip › File2/Confidence_High_Taxonomy/329236_out.krona.html]

Javascript must be enabled to view this page.

members
magnitude
magnitudeUnassigned
count
unassigned
taxon
rank

329236\_out


SRS893338\_contig\_number\_4105
1
5

superkingdom
2759
4

4751
kingdom
4

4
451864
subkingdom

4890
phylum
4

147537
subphylum
4

4
class
4891

4
4892
order

family
766764
2

1535326
genus
2


SRS1055040\_contig\_number\_contig-100\_977.978SRS893335\_contig\_number\_17814
species
5476
2

27319
family
2

2
genus
27320

2
species
73520

SRS1041116\_contig\_number\_1044SRS893366\_contig\_number\_26162
